# Impact of the COVID-19 Pandemic on Dermatology Practice Worldwide: Results of a Survey Promoted by the International Dermoscopy Society (IDS)

**DOI:** 10.5826/dpc.1101a153

**Published:** 2021-01-29

**Authors:** Claudio Conforti, Aimilios Lallas, Giuseppe Argenziano, Caterina Dianzani, Nicola Di Meo, Roberta Giuffrida, Harald Kittler, Josep Malvehy, Ashfaq A. Marghoob, H. Peter Soyer, Iris Zalaudek

**Affiliations:** 1 Department of Dermatology and Venereology, Dermatology Clinic, Maggiore Hospital, University of Trieste, Italy; 2 First Dermatology Department, Aristotle University of Thessaloniki, Greece; 3 Department of Dermatology, University of Campania, Luigi Vanvitelli, Naples, Italy; 4 Dermatology Section, Plastic and Reconstructive Surgery Unit, Campus Biomedico University, Rome, Italy; 5 Department of Clinical and Experimental Medicine, Dermatology, University of Messina, Italy; 6 Department of Dermatology, Medical University of Vienna, Austria; 7 Dermatology Department, Melanoma Unit, Hospital Clinic, University of Barcelona, Spain; 8 Dermatology Service, Department of Medicine, Memorial Sloan Kettering Cancer Center, New York, NY, USA; 9 The University of Queensland Diamantina Institute, The University of Queensland, Dermatology Research Centre, Brisbane, QLD, Australia; 10 Department of Dermatology, Princess Alexandra Hospital, Brisbane, QLD Australia

**Keywords:** COVID-19, teledermatology, survey, SARS-CoV-2, dermatology

## Abstract

**Introduction:**

The International Dermoscopy Society (IDS) conducted an online survey to investigate the impact of coronavirus disease 2019 (COVID-19) outbreak on the daily practice of dermatologists working with skin cancer patients, to collect data regarding the frequency of skin manifestations noticed by the members, and to obtain information about the use of teledermatology during the pandemic.

**Methods:**

All IDS members were asked to fill in a questionnaire, sent by email. A questionnaire available in English was sent to all IDS members (≈16.0000 members) by email. The questionnaire was anonymous, with a compiling time of less than 5 minutes. The survey was open for 30 days (from April 24, 2020 to May 24, 2020) and it could only be filled out once.

**Results:**

Overall, 678 dermatologists responded to the questionnaire; 334 members stated that there has been a reduction of more than 75% in daily work activity during the pandemic, 265 dermatologists worked fewer days per week, and 118 experienced telemedicine for the first time. Acrodermatitis was the most frequently observed skin manifestation (n = 80) followed by urticarial rash (n = 69), morbilliform rash (n = 53) and purpuric manifestation (n = 40). In regard to the role of teledermatology, 565 dermatologists reported an increased number of teleconsultations, and the number of melanomas diagnosed during the pandemic was practically 0 for 385 (56.78%) of respondents.

**Conclusion:**

This survey highlights that the outbreak had a negative impact on most dermatology services, with a significant reduction in consultation time spent for chronic patients, and an increased risk of missed melanoma and nonmelanoma skin cancer (NMSC) diagnosis. Moreover, our study confirms earlier findings of a wide range of skin manifestations associated with COVID-19.

## Introduction

On December 2019, a novel coronavirus, SARS-CoV-2, started the outbreak of coronavirus disease 2019 (COVID-19) that rapidly spread worldwide and caused thousands of infections and deaths. Because of the enormous burden, it was officially recognized as a pandemic by the World Health Organization on March 11, 2020 [1,2].

To cope with this worldwide emergency, hospitals have been rapidly reorganized to increase the number of intensive or sub-intensive care units, and other departments have been closed or readapted to focus only on urgent health care services, including non-deferrable dermatological visits. Moreover, telemedicine services were rapidly implemented mostly to guarantee the remote management of patients requiring continuous treatment [3,4]. An increased number of unusual dermatological manifestations were reported, initially among children and later in adults, which raised the suspicion of a possible correlation between coronavirus disease 2019 (COVID-19) and dermatological eruptions [5–7]. To date, no skin manifestation appears pathognomonic of COVID-19, as many of the described skin rashes can be seen during the course of other viral infections. For this reason, the International Dermoscopy Society (IDS) conducted an online survey with 3 aims: first, to investigate the impact of the COVID-19 outbreak on the daily practice of dermatologists working with skin cancer patients; second, to collect data regarding the frequency of skin manifestations noticed by its members; and third, to obtain information about the use of teledermatology during the pandemic.

## Methods

A questionnaire was sent to all IDS members (16,0000 members) by email. It consisted of 24 questions, 4 regarding the respondent’s personal data, 7 on the organization of the dermatology department/clinic and on possible contact with COVID-19 positive patients, 9 on cutaneous manifestations in COVID-19 patients, and 4 on teledermatology and diagnosis during the pandemic.

The questionnaire was anonymous, of 5-minute duration, and was approved by IDS Executive Board members. The survey was open for 30 days (from April 24, 2020 to May 24, 2020). The answers were saved only for completed questionnaires on on the digital platform SurveyMonkey (SurveyMonkey, San Mateo, CA, USA) to which only 3 members (CC, AL, IZ) had access to monitor the progress of the survey.

## Results

Overall 678 dermatologists from 52 different countries completed the questionnaire (418/678 women, 61.65%; 260/678 men, 38.35%; mean age 47.7; range 26–76 years). Worldwide distribution of participants involved all 5 continents, and the majority worked in private practice (n = 264, 38.94%) clinics or university hospitals (n = 232, 34.22%) (Table 1).

Dermatologists, 334/678 (49.26%), stated that since the beginning of the pandemic, there has been more than 75% a reduction in daily work activity to avoid crowding and to focus on dermatological emergencies. To prevent febrile patients from entering the hospital, a dedicated triage was created in most of the centers (376/678; 55.46%) where dermatologists worked to measure body temperature and to equip patients with surgical masks (Table 2). To cope with the emergency, the majority of participants 265/678 (39.08%) stated that they worked fewer days per week at the dermatology departments to ensure urgent or non-renewable visits, 9/678 (21.98%) continued working on a daily basis, and 76/678 (11.21%) worked actively in COVID-19 dedicated departments. Among respondents, 188/678 dermatologists (27.73%) performed smart working performing visits through digital platforms and experienced telemedicine for the first time.

Overall, 214 dermatologists stated to have had contact with confirmed (87; 12.83%) or strongly suspected (124; 18.29%) COVID-19 patients. Of those 214 dermatologists, only 24 (3.54%) tested positive for SARS-CoV-2. Less than half of respondents (n = 297; 43.81%) reported the availability of sufficient personal protective equipment.

Regarding specific questions on dermatological manifestations of the virus, 1/6 of respondents stated to have visited COVID-19 positive (confirmed PCR on nasopharyngeal swab) patients with clinical manifestations (Table 3). Most clinical manifestations were observed in patients aged 1–20 years (n = 58, 31.44%), followed by 20- to 40-year age group (n = 54, 29.34%), 40–60 years (n = 42; 22.82%), and older than 60 (n = 30; 16.40%).

The most frequently noted clinical manifestations were acrodermatitis (n = 80; 21.50%), urticarial rash (n = 69; 18.54%), morbilliform skin rash (n = 53; 14.24%), purpuric manifestations (n = 49; 13.17%), followed by erythema polymorphous like rash (n = 37; 9.94%) and chickenpox like rash (n = 30; 8.06%) (Table 3). The skin lesions either appeared after the onset of systemic symptoms (n = 75; 35.89%) or were the only clinical manifestation in asymptomatic patients (n = 57; 27.27%) (Table 3). Most dermatologists reported that skin manifestations were mostly observed in asymptomatic (n = 89; 52.67%) or paucisymptomatic patients (n = 55; 32.54%).

The last part of the questionnaire (questions 21–24) focused on the implementation of telemedicine during the pandemic. Respondents also noted that the number of unofficial teleconsultations (eg, by mail, SMS, Skype, WhatsApp, messenger, etc.) that they were asked to do during the COVID-19 pandemic has increased by 83.33% (n = 565).

Remarkably, the number of melanomas diagnosed in these months was practically 0 for more than half (n = 385; 56.78%) of respondents. The reduction in diagnosis is also superimposable for nonmelanoma skin cancers (NMSCs) for which 253 dermatologists stated that the diagnosis was very close to 0, and 184 reported that the NMSC diagnosis was 50% less compared to previous months. Last, during the pandemic, the main reason for dermatological examinations were tumors considered by the patient to be suspicious, pruritic eruptions, followed by chronic skin diseases.

## Discussion

The IDS promotes research and education of dermoscopy, a tool that is mostly implemented in early skin cancer diagnosis. In fact, the majority of IDS members work, at least to some extent, in skin cancer care. The fact that nearly 700 colleagues from 52 countries responded to the survey allows a proper view on the effective global impact of the COVID-19 pandemic on the routine work in dermatological care. Our data highlight a 75% reduction of the work and suggest that the COVID-19 health emergency had a particularly negative impact on dermatological care and practice [8]. This can be explained by the fact that most private and public clinics started to postpone all nonurgent visits and elective surgeries and medical procedures in order to reduce the risk of nosocomial transmissions. Although oncologic visits or surgery were guaranteed throughout the pandemic, our survey suggests that there was a significant decrease in daily clinical care of skin cancer patients. The survey revealed an alarming decrease of melanoma diagnosis during the pandemic. It is important to monitor whether this will induce an increase of thicker melanomas in the forthcoming months and years. This is especially relevant for fast-growing melanomas that have been estimated to invade the dermis at a rate of approximately 0.5 mm per month [9]. To overcome this challenge, it is paramount to provide timely care to patients, especially those who have a high risk of developing melanoma. Moreover, it would be essential, now more than ever, to increase patient and general practice physician education on early melanoma detection [10]. In this regard, the fight against skin cancer in the COVID-19 era should be largely based on 2 important tools, skin self-examination and teledermatology.

While there was a worrisome decrease in melanoma or NMSC diagnosis during the months of lockdown, 111/678 participants stated to have seen skin manifestations in confirmed or suspected COVID patients, obtaining real-life data quickly from all over the world about the skin manifestations of the SARS-CoV-2 virus. A majority of cutaneous manifestations of COVID-19 have been observed in asymptomatic or symptomatic patients, which means that dermatological clues may help clinicians suspect a COVID-19 infection when the symptoms cannot be associated with other causes. The urticarial and morbilliform rash were the most frequent signs followed by acrodermatitis (chilblains); the latter is not typical of the summer months and it should therefore be a warning to suspect COVID-19 infection, especially in the pediatric age. Other authors have also shown that this sign is more frequent in children [11].

Finally, our survey highlights that teledermatology consultations strongly increased during the outbreak. Data from this survey demonstrated that, since the beginning of the pandemic, worldwide outpatient activities have been reduced and online dermatological consulting has increased significantly. Online consultation can easily be used for dermatological diagnosis, as an initial triage of inflammatory pathologies or suspicious lesions can be identified via a video call with the aim of establishing the need to perform a dermoscopic examination to exclude or confirm a diagnosis.

During this time of unexpected and sudden changes and the need of social distancing, teledermatology has made it possible for many dermatologists to take care of patients remotely [12,13], and this tool is certain to be used increasingly in the immediate future. According to the recommendations from the European Academy of Dermatology and Venereology Task Forces, this is the time to organize and implement teledermatology services [14], in order to manage and care patients with skin lesions that are suspected of malignancy, non-melanoma skin cancer candidates to medical therapy and/or acute or chronic inflammatory cutaneous disorders from the safety of their homes. However, lesions suspected to be malignant should preferentially be managed via face-to-face visit [15].

This study has several limitations to underline: first of all, although the IDS community has almost 16,000 members, the response rate was 4.2% (678 responses/16,000 members) despite using a mailing list to reach as many members as possible. At first, it was decided to use 2 versions of the questionnaire, one in English and one in Russian; however, in order to avoid terminological bias and to ensure homogeneity of the data collected, only the questionnaires obtained in English were considered, considering that the official language of the IDS is English. Another limitation was that the terms “acrodermatitis’” and ‘”chilblains” were used synonymously because in the first weeks of the outbreak, when acral manifestations were observed in the pediatric population with the viral infection, there was still no direct correlation with either one of the 2 manifestations.

## Conclusions

In conclusion, our study highlights that the outbreak had a negative impact on most dermatology services with a significant reduction in the time spent with patients with chronic disease and with a currently undefined risk of missed melanoma and NMSCs. Moreover, our study confirms earlier findings a wide range of skin manifestations associated with COVID, whereby especially the younger population develops skin lesions.

Following recommendations may help to close the gap of delayed healthcare for dermatologic patients in the next months: (i) improve accessibility to dermatologic care and the public health service system; (ii) implement prevention campaigns for the early detection of skin cancers; (iii) reactivate the follow-up of patients with melanocytic and nonmelanocytic tumors through reminders (calls, emails) to facilitate diagnostic and therapeutic adherence; and (iv) acrodermatitis, urticarial rash, and morbilliform skin rashes should lead to suspicion of COVID-19 infection.

## Figures and Tables

**Table 1:** Characteristics of the 678 Members Who Completed the International Dermoscopy Society Survey

**Sex**	
Men	260 (38.35%)
Woman	418 (61.65%)
Mean age	47.7 (range 26–76)
**Workplace (more than 1 answer possible)**	
University hospital	232 (27.79%)
Private hospital	67 (8.02%)
Private clinic	264 (31.62%)
Public clinic	127 (15.20%)
Outpatient clinic	145 (17.37%)

**Table 2:** Answers from the International Dermoscopy Society Survey Questions

	N=678	
	%	Count
**Did you reduce your daily work activity since the beginning of the pandemic?**		
Yes, about 25%	10.17%	69
Yes, about 50%	18.44%	125
Yes, about 75%	18.44%	125
Yes, more than 75%	49.26%	334
No	3.69%	25
**Was there a dedicated triage for dermatological outpatients?**		
Yes, patients wear the mask and temperature was measured	35.40%	240
Yes, without measuring the temperature	20.06%	136
No, but patients wear the mask	22.42%	152
None of the above	22.12%	150
**Did you work in COVID-19 dedicated departments?**		
Yes, not as dermatologist	11.21%	76
No, I worked only using teledermatology	27.73%	188
No, I worked every day as dermatologist	21.98%	149
No, I worked few days per week in the dermatology department	39.08%	265
**Did you come into contact with COVID-19 positive patients?**		
Yes, I got in contact with confirmed case	12.83%	87
Yes, I got in contact with patient confirmed case	18.29%	124
No	68.88%	467
**Did you test positive for COVID-19?**		
Yes	3.54%	24
Not	43.81%	297
A test has not been performed	52.65%	357
**Did you see COVID-19 patients with skin manifestations?**		
Yes	16.37%	111
No	83.63%	567
**What was the average age of the patient(s)? (more than 1 answer possible)**		
1–20	31.44%	58
20–40	29.34%	54
40–60	22.82%	42
>60	16.40%	30
**The number of teleconsultations you were asked to do during COVID-19 pandemic was**		
As usual	16.67%	113
Increased a little bit	23.30%	158
Doubled	16.96%	115
Multiplied	43.07%	292
**The number of melanomas you diagnosed (including by teleconsultations) during the pandemic was**		
The same as usual	15.93%	108
A little bit lower than usual	9.89%	67
Almost half of the usual	4.72%	32
Less than half of the usual	12.68%	86
Almost zero	56.78%	385
**The number of nonmelanoma skin cancers you diagnosed (including by teleconsultations) during the pandemic was**		
The same as usual	16.52%	112
A little bit lower than usual	10.77%	73
Almost half of the usual	8.26%	56
Less than half of the usual	27.11%	184
Almost zero	37.31%	253
**During the pandemic, what was the main reason prompting patients to seek live dermatologic consultation? (more than 1 answer possible)**		
Tumors	39.09%	265
Chronic skin diseases	26.99%	183
Pruritic eruptions	37.32%	253
Cosmetic problems	8.55%	58
No predominant reason	24.19%	164

**Table 3:** Skin Findings in Patients Positive for SARS-CoV-2 Infection According to International Dermoscopy Society Survey Questions

What kinds of skin manifestations did you see? (more than 1 answer possible)	%	Count
Morbilliform skin manifestation	14.24%	53
Erythema polymorphous like rash	9.94%	37
Erythrodermic rash	3.24%	12
Chicken-pox like rash	8.06%	30
Urticarial rash	18.54%	69
Purpuric manifestation	13.17%	49
Acrodermatitis (chilblains)	21.50%	80
Herpes zoster virus reactivation	4.60%	17
Psoriatic flare	2.41%	9
Atopic flare	4.30%	16
**Skin manifestations (more than 1 answer possible)**		
Anticipated systemic symptoms	19.14%	40
Appeared after systemic symptoms	35.89%	75
Were the only ongoing manifestation of illness	27.27%	57
Were a tardive manifestation of illness	17.70%	37
**Skin manifestations were more commonly seen in (more than 1 answer possible)**		
Asymptomatic patients	52.67%	89
Paucisymptomatic patients	32.54%	55
Symptomatic patients	14.79%	25
